# Union of Brodsky type 1/Eichenholtz stage III Charcot neuroarthropathy after forefoot arthrodesis

**DOI:** 10.1016/j.ijscr.2020.04.040

**Published:** 2020-05-11

**Authors:** Ananto Satya Pradana, Krisna Yuarno Phatama, Adhi Satrio Utomo, Muhammad Hilman Bimadi, Marvin Anthony Putera, William Putera Sukmajaya, Edi Mustamsir, Mohamad Hidayat

**Affiliations:** Department of Orthopaedics and Traumatology, Saiful Anwar Hospital-Brawijaya University, Malang, Indonesia

**Keywords:** Charcot neuroarthropathy, Diabetes mellitus, Forefoot arthrodesis, Surgical intervention, Case report

## Abstract

**Introduction:**

One of the surgical intervention options for Charcot neuroarthropathy (CN) is arthrodesis. The arthrodesis procedure for the foot and ankle joint have been widely used in previous studies. This study aimed to evaluate the functional and radiological outcomes after arthrodesis procedure for the CN patient with Brodsky type 1/Eichenholtz stage III.

**Presentation of case:**

A 49-years-old diabetic woman presented with alteration of her right foot’s shape and a cracking sensation while walking in the last six months. Rocker-bottom deformity and a decreased sensation on the right foot were found, and the initial American Orthopaedic Foot & Ankle Society (AOFAS) score was 45. Subsequently, forefoot arthrodesis of the right foot was performed.

**Discussion:**

Several studies state that arthrodesis procedure is often used for CN management in order to achieve a plantigrade and stable foot. This study presents an improvement of the foot arch, AOFAS score, and union of the talus six months after surgery.

**Conclusion:**

The arthrodesis procedure by using screws and Kirschner wire (K-wire) fixation is an effective method in CN management if the patient is compliant. This study showed a good result, anatomically restored the foot arch, and excellent radiological union, but different tools to analyze foot functional status and longer follow up period are needed for a better analysis.

## Highlights

• Forefoot arthrodesis by using screws and Kirschner wire fixation could achieve a satisfactory bony union.
• AOFAS score of the right foot gradually improved from 45 to 86 in six months period after arthrodesis.
• There was an improvement of right foot’s Meary’s angle from 22° to 8° six months after surgery.
• The proper diagnosis and treatment of CN could prevent ulceration and lower extremity amputation.
• Continuous follow-up and patient compliance are essential to minimize the complications of arthrodesis.

## 1 Introduction

Charcot neuroarthropathy (CN) is a progressive and destructive condition of bones, joints, and soft tissues [1,2]. Jean-Martin Charcot first described CN in 1868 [3], while Jordan was the one who first mentioned the relationship between CN and diabetes mellitus (DM) [4]. There are several available CN classifications: Brodsky (anatomical classification) and Eichenholtz (clinical and radiological classification) [5]. The most predominant area of CN are the foot and ankle [2]. The total incidence and prevalence are between 0.1%–29% and 0.08–13% among diabetic patients, and occurrence of amputation in CN with ulceration was as high as 15%–67% [6,7]. Earliest manifestations of CN are similar to simple sprain, deep vein thrombosis, osteomyelitis, cellulitis, and rheumatoid arthritis [5]. Misdiagnosis and improper treatment could lead to limb-threatening condition and amputation caused by foot deformity and ulceration [1,2]. Rocker-bottom deformity, described as midfoot collapse and deformity, which is typical for the chronic CN [8]. Subsequently, the quality of life on diabetic patients with CN is generally weaker compared to those without [2,5]. However, both non-surgical and surgical treatments are available for CN management, with the main goal to obtain a stable and plantigrade foot free of ulcerations [7]. The current treatment of choice for Eichenholtz stage III CN is reconstruction with internal fixation [5]. This case report aims to evaluate the functional and radiological outcomes after arthrodesis procedure in a CN patient with Brodsky type 1/Eichenholtz stage III. The work has been reported in line with the SCARE criteria [9].

## 2 Presentation of case

A 49-years-old woman presented with alteration of her right foot shape, cracking sensation, and progressing difficulty while walking in the last six months. She never consulted any physicians about her right foot before. There was no history of trauma and infection, but there was a history of uncontrolled type 2 DM in the last two years. She did not consume her oral antidiabetic drugs routinely; her diabetic medication was switched to insulin therapy in the last six months to better control her disease.

**Fig. 1:**
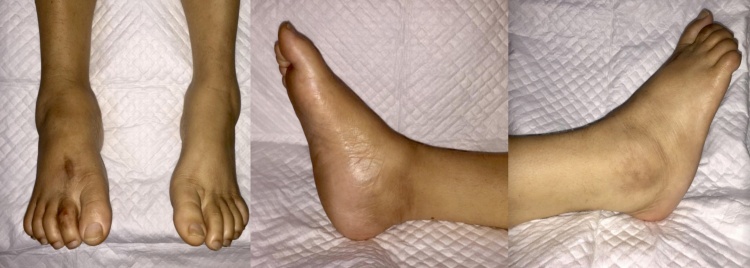
The clinical picture showed a rocker-bottom foot and the disappearance of foot tripod on the right foot.

**Fig. 2:**
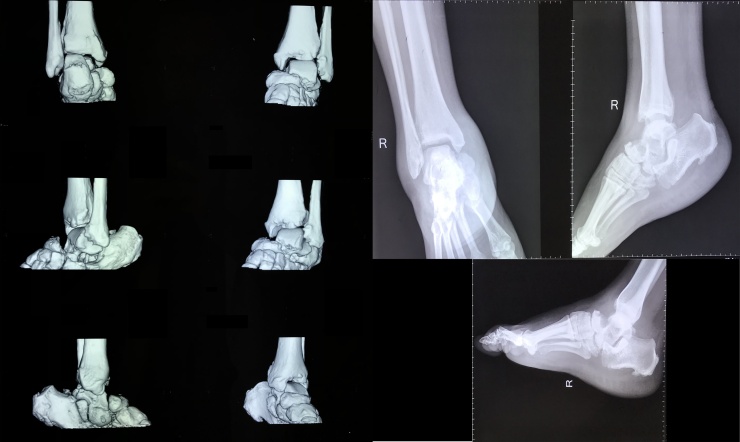
Radiographic result of the right foot demonstrated a deformity of the forefoot bone.

Physical examination revealed rocker-bottom deformity, but there was no edema, ulceration, or local rise in temperature (Fig. 1). There was a foot tenderness with visual analog scale (VAS) score of 2–3, a decrease of distal sensory perception, a capillary refill time of less than 2 seconds, and limited ankle range of motion (ROM). CT-scan and plain radiographs revealed the bone deformity of the forefoot with Meary’s angle of 22°, Bohler angle of 88°, and Gissane angle of 125° (Fig. 2).

**Fig. 3:**
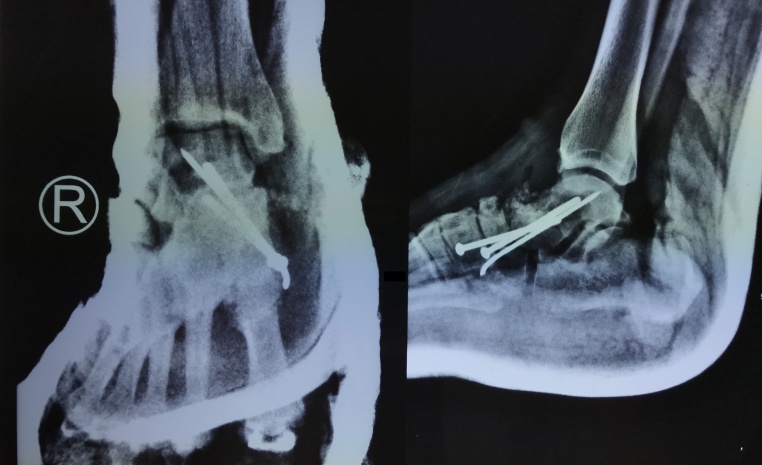
Plain radiograph after forefoot arthrodesis procedure fixed by screws and K-wire.

**Fig. 4:**
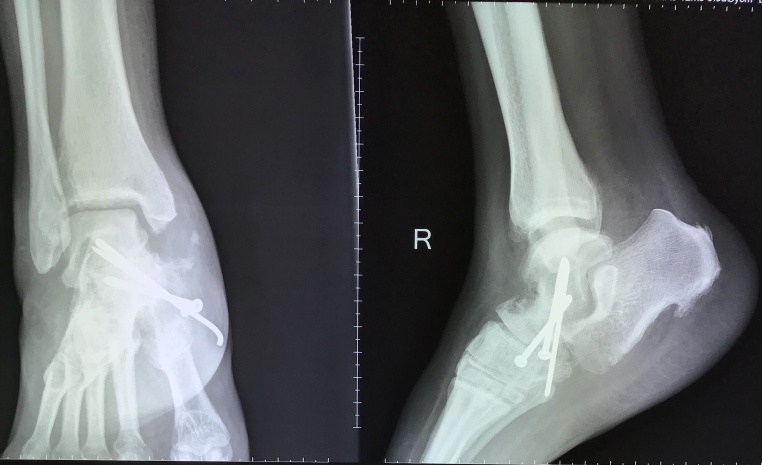
Plain radiograph in three months after forefoot arthrodesis showed the bony union of the talus bone fixed by screws and K-wire.

**Fig. 5:**
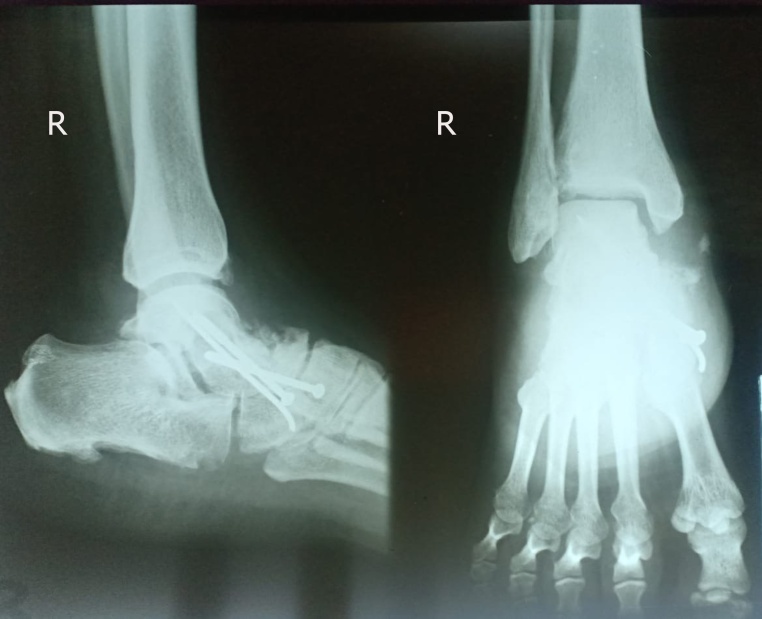
Plain radiograph in six months after forefoot arthrodesis showed the bony union and good improvement of Meary’s angle measurement at 8° from 22° before surgery.

After establishing the diagnosis, the patient underwent forefoot arthrodesis by using screws and K-wire fixation (Fig. 3). The initial AOFAS score was 45 out of 100, then two months after surgery the score increased to 61. Furthermore, the radiological union of talus was obtained three months after surgery (Fig. 4); the AOFAS score further improved to 69. The AOFAS score continued to increase in the fourth and sixth months follow-up after surgery with a score of 78 and 86, respectively. The foot pain was absent sixth months after the surgery while physical and radiological examination showed improvement of the foot arch and the Meary’s angle improved to 8° (Fig. 5). The patient was compliant to post-surgical advices. Thus, there was no complication, and there was an improvement of her overal quality of life.

## 3 Discussion

CN is a disabling complication of DM [10], and one of the most challenging clinical problems for foot and ankle surgeon [11]. CN substantially affects the foot and ankle. In Brodsky type 1, 60%–70% of cases involves the tarsometatarsal or naviculocuneiform joints. The forefoot is the most common area of ulceration in the diabetic foot. Garapati et al. reported that 74% of all diabetic foot ulcers were located under the metatarsal heads [12]. Many risk factors are related to CN: leprosy, alcoholism, syphilis, syringomyelia, rheumatoid arthritis, multiple sclerosis, and traumatic injury [13]. The progressive disease process is triggered by unrecognized repetitive trauma because of a loss of sensation and reactive hyperemia mechanisms [14]. The pathogenesis was still unclear, but it is believed the underlying pathological process is due to either neurotraumatic or neurovascular mechanism [15]. Limb-threatening condition could occur if the pathological process is left untreated, prompting lower extremity amputation [15].

Complete off-loading immobilization with total contact cast (TCC), Charcot restraint orthotic walker (CROW), bracing, and antiresorptive agents are applicable for the initial treatment of CN. These methods may control the harmful effect of trauma, but the damage caused by osteoclast activity will keep progressing [16,17]. When the non-surgical management fails or inapplicable for CN, surgery has an important role in limb preservation [7]. Described techniques consist of Achilles tendon lengthening, plantar osteotomy, osseous debridement, realignment osteotomy, selective or extended arthrodesis, and open reduction with various forms of internal fixation with or without external fixation. The primary indications for surgery are pain, recurrent ulceration, and deformity with instability; the goal of treatment is to obtain a plantigrade foot with a good and stable alignment [14].

Once CN is diagnosed, it mostly appears in an advanced stage, and it is hard to treat. Implant breakage is a common cause of treatment failure [3]. Thomas et al. reported asymptomatic non-union and partial implant breakage after triple arthrodesis in diabetic 50-years-old female (Eichenholtz stage III) with bilateral non-plantigrade foot deformity. Furthermore, a bilateral correction was performed and the result of podogram showed a plantigrade foot position without pathological weight-bearing at the midfoot area [4]. The combination of poor diabetic fracture healing with recurrent or progressive fracture patterns mostly occurs in CN. For this reason, fixation methods must consider the delay in bone healing, decreased bone mineral density, and potential loss of fixation when reconstructing foot and ankle deformities associated with CN [18].

The proper timing of surgical procedure for CN is still unclear [11,19], but it is believed that surgical procedure in the acute inflammatory phase of CN could lead to an increased risk of wound healing problems, difficult fixation, and even surgical failure [19]. Prior study by Lowery et al. described that arthrodesis is the most common procedure to treat CN with a fusion of 76% [19]. Furthermore, the total cost of arthrodesis procedure is believed to be at least 14% less than the total cost of below the knee amputation. Simon et al. also reported that the mean charge for arthrodesis procedure and follow up was $13.511 compared with $25.090 for extremity amputation [11]. In line with the previous statement, the cost-effectivity was one of our considerations when educating the patient to undergo surgical procedure. Thus, the patient would be willing to undergo surgical procedure to obtain a better quality of life. The forefoot arthrodesis by using screws and K-wire fixation was proven to prevent deformity progression in this study. Furthermore, the procedure succeded to achive plantigrade foot with excellent function (final AOFAS score of 86). However, the patient's post-operative compliance was also an important factor in ensuring a satisfactory outcome.

Despite all the positive results, this study had some limitations due to the short follow-up period and only AOFAS scoring system was used to evaluate the post-operative functional outcome. Nevertheless, this present study showed results which may be valuable for future studies.

## 4 Conclusion

The arthrodesis procedure by using screws and K-wire fixation is an effective method in CN management, resulting in anatomical foot arch and radiological union. The patient's post-operative compliance is also an important determining factor which affects the overall outcome.

## Conflicts of interest

The authors declared that there is no conflict of interest regarding this study.

## Sources of funding

The authors solely funded this study.

## Ethical approval

The study has been approved by the Ethical Committee of Medical Research of Saiful Anwar General Hospital, Malang, East Java, Indonesia. This study is in accordance with Declaration of Helsinki.

## Consent

Informed consent has been obtained from the patient.

## Author contribution

Ananto Satya Pradana: conceptualization, writing original draft preparation, supervision.

Krisna Yuarno Phatama: conceptualization.

Adhi Satrio Utomo: data collecting and data interpretation.

Muhammad Hilman Bimadi: literature search, writing the paper and editing.

Marvin Anthony Putera: writing the paper and editing.

William Putera Sukmajaya: writing the paper and editing.

Edi Mustamsir: conceptualization.

Mohamad Hidayat: conceptualization.

## Registration of research studies

This study is a case report and does not registered in human studies research registry.

## Guarantor

Ananto Satya Pradana, MD.

## Provenance and peer review

Not commissioned, externally peer-reviewed.
